# A deep learning-based approach for the detection of cucumber diseases

**DOI:** 10.1371/journal.pone.0320764

**Published:** 2025-04-11

**Authors:** Lars Raufer, Jasper Wiedey, Malte Mueller, Pascal Penava, Ricardo Buettner

**Affiliations:** 1 Chair of Information Systems and Data Science, University of Bayreuth, 95447 Bayreuth, Germany; 2 Chair of Hybrid Intelligence, Helmut-Schmidt-University / University of the Federal Armed Forces Hamburg, 22043 Hamburg, Germany; Universitat Jaume 1, SPAIN

## Abstract

Cucumbers play a significant role as a greenhouse crop globally. In numerous countries, they are fundamental to dietary practices, contributing significantly to the nutritional patterns of various populations. Due to unfavorable environmental conditions, they are highly vulnerable to various diseases. Therefore the accurate detection of cucumber diseases is essential for maintaining crop quality and ensuring food security. Traditional methods, reliant on human inspection, are prone to errors, especially in the early stages of disease progression. Based on a VGG19 architecture, this paper uses an innovative transfer learning approach for detecting and classifying cucumber diseases, showing the applicability of artificial intelligence in this area. The model effectively distinguishes between healthy and diseased cucumber images, including *Anthracnose, Bacterial Wilt, Belly Rot, Downy Mildew, Fresh Cucumber, Fresh Leaf, Pythium Fruit Rot*, and *Gummy Stem Blight*. Using this novel approach, a balanced accuracy of 97.66% on unseen test data is achieved, compared to a balanced accuracy of 93.87% obtained with the conventional transfer learning approach, where fine-tuning is employed. This result sets a new benchmark within the dataset, highlighting the potential of deep learning techniques in agricultural disease detection. By enabling early disease diagnosis and informed agricultural management, this research contributes to enhancing crop productivity and sustainability.

## Introduction

Agriculture is a vital pillar of every economy, significantly influencing a country’s economic development [1,2]. It not only generates essential raw materials and food products but also creates numerous job opportunities [3]. However, agriculture faces myriad challenges, such as irregular rainfall and poor soil fertility [4]. Among these challenges, one issue stands out prominently: the infestation of crops by diseases [5]. Plant diseases significantly impact global agricultural productivity, leading to yield losses and economic damage [6,7]; for instance, they are responsible for a loss of 35% of crop yields in India [8]. Cucumbers are one of the most important greenhouse crops in Europe, as well as in the Near and Far East [9]. Global cucumber production amounted to 88 million tons in 2019, 80% of which is produced in China [10]. A large proportion of production also takes place in Iran, Turkey, the USA, Japan, and Russia [9]. In these and many other countries, cucumbers are an important part of society’s nutrition [11]. They are particularly susceptible to various diseases caused by adverse environmental conditions [6]. These diseases, including *Anthracnose, Bacterial Wilt* and *Downy Mildew*, can cause considerable harm and create gaps in food supply chains [6,12]. *Downy Mildew*, for example, can lead to yield losses up to 80%, as well as seed losses of up to 70% [13]. Early detection and treatment are crucial to improve crop yields and support farmers, especially in regions like Bangladesh, where the used dataset was collected [14]. By diagnosing the disease at an early stage, which is essential as fruit and vegetables spoil quickly, farmers can reduce their use of pesticides in the long term to increase the quality and sustainability of their harvest [15]. The global use of pesticides totaled 4.1 million tons per year in 2017 [16]. Pesticides negatively affect people’s health and promote the development of diseases such as cancer [17]. In addition, the overuse of pesticides leads to pests developing resistance, which means that even stronger chemicals must be used [18].

**Fig 1:**
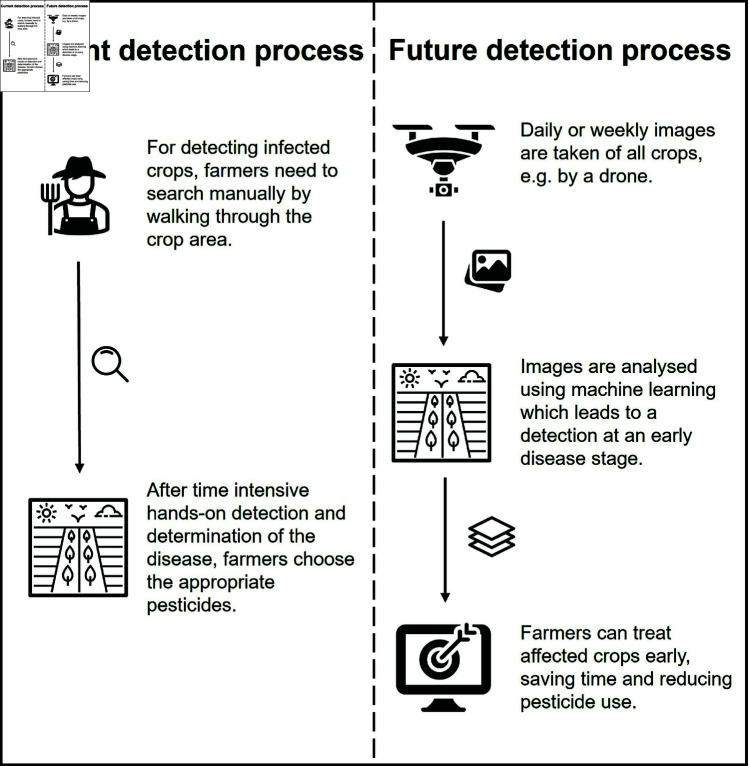
Use case: Detecting cucumber diseases using machine learning.

For this reason, there is an urgent need for efficient and accurate disease detection methods, given the importance of early diagnosis for plant diseases [19]. Traditional methods are often time-consuming and inaccurate, highlighting the increasing focus on automated solutions like machine learning and deep learning technologies [6,12]. Human diagnosis of diseases is prone to errors and can lead to diseases going undetected [20]. This can endanger the entire crop [21]. Machine learning models are faster and more precise [20]. They detect diseases earlier, which allows farmers to respond in a timely and appropriate manner [22]. This saves labor costs as well as time, as illustrated in Fig 1, comparing the current detection process with the future detection process [23]. Moreover, new diseases regularly emerge due to climate changes [24]. For farmers, it is nearly impossible to discern all diseases [25]. This is why the use of modern technologies is advisable [20]. However, in the application of image processing for plant disease detection, the absence of large-scale datasets outside laboratory settings presents a significant obstacle [8].

Existing plant disease detection technologies, for example, use a MobileNet V3 network that is specially optimized for mobile devices [26]. However, similar procedures, although they achieve good results, are still underdeveloped and often expensive [26]. Plant photographs for disease detection are frequently of poor quality due to close planting [27]. Traditional methods based on human inspection are inefficient, particularly in the early stages of disease [26,28]. Developing a machine learning model based on a specialized dataset that displays cucumber diseases in a hyperspectral array of images is therefore crucial [6]. Many recognition and diagnostic methods follow the pipeline method of image segmentation, feature extraction and pattern recognition. However, two problems arise when using the pipeline method: First, the accuracy of such methods is highly dependent on how the visible disease features are selected and extracted. Moreover, the complexity of these approaches is notably increased due to the variable lighting conditions often encountered in field images [7]. Research has also shown that the background of images significantly influences detection and that identifying diseases from a single lesion in an image can alleviate the problem of data scarcity for training deep learning models [29].

Previous studies have demonstrated the effective application of deep learning techniques in object recognition, particularly in the identification of cucumber leaf diseases [7,14,28,30–38]. Table 1 shows a significant limitation of current studies. Models often operate with a limited number of classes, focus either exclusively on the cucumber fruit or its leaves and don’t distinguish between healthy specimens and those affected by diseases, only identifying the type of disease. Building upon this foundation, our work contributes by presenting a model capable of discerning the health status of cucumbers and their leaves, as well as identifying specific diseases, showing that machine learning and deep learning are valid options for these cases. Employing a transfer learning approach with the pre-trained VGG19 model, we anticipate our model to yield accurate outcomes conducive to effective disease management. As part of our methodological innovation, we modified the traditional transfer learning approach by making the last eight layers of the model adjustable from the start of the training process, rather than fine-tuning them, in order to optimize discerning the health status of cucumbers and their leaves. This innovative technique reduces training complexity by focusing adjustments on the upper layers, enabling more effective and targeted optimization.

**Table 1:** Concept matrix of related work.

Reference	Year	Research subject	Image acquisition	Number of classes	Methodology	Accuracy	Limitation
Lin et al. [31]	2019	Identification and quantification of Powdery Mildew on cucumber leaves	Acquisition under laboratory conditions (total of 50)	2	CNN	96.08%	Laboratory conditions
Ozguven [32]	2020	Detection and quantification of mildew disease on cucumber leaves	Acquisition under natural conditions (total of 175)	5	R-CNN	94.86%	Focus solely on mildew disease
Bansal et al. [30]	2023	Detection of severity level of cucumber leaf spot disease	Acquisition under natural conditions (total of 50,000)	5	ResNeXt50	97.81%	Focus solely on cucumber leaf spot disease
Zhang et al. [33]	2021	Identification of cucumber leaf diseases	Acquisition under natural conditions	3	InceptionV3	96.11%	Small number of classes
Hussain et al. [28]	2021	Multiclass cucumber leaf disease recognition	Acquisition under natural conditions	5	VGG & InceptionV3	96.50%	Focus solely on cucumber leaves
Khan et al. [34]	2021	Cucumber leaf disease recognition	Acquisition under natural conditions	7	DenseNet201	98.40%	Focus solely on cucumber leaves
Ma et al. [7]	2018	Detection of cucumber leaf diseases	Acquisition under natural conditions	4	DCNN	93.40%	Small number of classes
Omer et al. [35]	2022	Diagnosis of cucumber leaf diseases	Acquisition under natural conditions (total of 4,868)	6	CNN	98.19%	Focus solely on cucumber leaves
Nguyen & Nguyen [36]	2023	Identification of abnormal cucumber leaves	Acquisition under natural conditions (total of 154)	4	U-Net	96.33%	Small number of classes
Tani et al. [37]	2018	Diagnosis of multiple cucumber leaf infections	Acquisition under natural conditions (total of 48,311)	25	VGG-Net	95.50%	Focus solely on cucumber leaves
Jeny et al. [38]	2021	Cucumber disease recognition and classification	Combination of images in field conditions and open source images (total of 521)	5	Random forest	85.84%	Insufficient classification accuracy
Mia et al. [14]	2021	Cucumber disease and cucumber leaf disease recognition	Acquisition under laboratory and natural conditions (total 525)	6	Random forest, MobileNetV2	89.93%, 93.23%	Insufficient classification accuracy
Junior & Majiid [39]	2023	Cucumber disease and cucumber leaf disease detection	Acquisition under natural conditions (total of 1,280)	8	EfficientNetB0, ResNet50	94.98%, 89.57%	Insufficient classification accuracy

Our objective was to develop a precise, efficient and comprehensive solution for the detection of cucumber diseases, addressing the critical necessity for early diagnosis of plant diseases through image classification. Consequently, the primary contributions of this study are as follows:

- We develop a deep learning model regarding the detection of the health status of cucumbers and their leaves, as well as identifying specific diseases with an accuracy of 95.31% outperforming the current benchmark of 94.98% set by Junior and Majiid [39].
- We introduce an innovative approach by making the last eight layers adjustable already from the beginning of the training phase, achieving a balanced accuracy of 97.66%, which surpasses previous methods and demonstrates the effectiveness of this technique in disease detection.

The paper is structured as follows: The next section provides the theoretical background regarding the diseases mentioned in the dataset and current approache to cucumber disease detection. Subsequently, we present a detailed description of our methodological approach followed by the results. After that, we discuss their implications and draw a conclusion that contains the limitations of our work and propose possible future research directions.

## Research background

### Relevant diseases and treatment in cucumber farming

In order to identify the most relevant and common cucumber diseases, research was carried out and existing data sets were analyzed. This resulted in the following diseases: *Anthracnose, Bacterial Wilt, Belly Rot, Downy Mildew, Pythium Fruit Rot* and *Gummy Stem Blight* [6].

*Anthracnose* commonly affects cucumbers as a fungal ailment [40]. Throughout the growing phase, this condition can lead to the rotting of the fruit, canker on stems, spotting on leaves and blight affecting all parts of the cucumber plant that are exposed to the air [6]. During warm and moist summer seasons, severe outbreaks of this disease can result in premature leaf shedding, diminished crop yields and a decline in the quality of the fruits [6,41]. The most commonly observed signs of this disease in agricultural fields are spots on the leaves [42,43]. The fungicidal active ingredient Fludioxonil is effective in the treatment of *Anthracnose* [44]. According to Li et al. [44], the addition of certain adjuvants to fludioxonil improves the effectiveness and efficacy of the fungicide in the treatment of diseased cucumbers. Parada et al. [45] have shown that fresh mushroom substrate made from the mushroom Hatakeshimeji and its water extract is also highly effective against *Anthracnose*.

*Bacterial Wilt* is a common disease affecting cucumbers [46]. The primary symptom of this disease is wilting, which eventually results in the plant’s death [47]. As the infection advances, leaves may develop yellowing (chlorosis) and dead (necrotic) areas [6]. Typically, symptoms swiftly spread along individual vines, moving towards the base of the plant (crown) and ultimately leading to the death of the whole plant [6,47]. The management of *Bacterial Wilt* primarily focuses on preventive strategies, including the utilization of healthy seeds, the eradication of infected plant matter, the sterilization of tools and the selection of more resistant varieties of plants [48]. Although chemical pesticides are applied, their efficacy may be diminished due to the pathogen developing resistance [49].

*Belly Rot* in fruits is caused by the fungus Rhizoctonia solani [6,50]. This disease manifests on cucumbers as water-soaked lesions that are tan to brown in color, typically appearing at the blossom end and underside of the fruit [6,30,50]. As belly rot progresses, these lesions become swollen and crater-like, adopting an irregular shape and eventually drying up [6,50]. The treatment of *Belly Rot* in cucumbers can be challenging [51]. Traditional methods such as fungicides often do not provide sufficient control [51]. Research is investigating biological control methods with antagonistic bacteria and fungi, but these may show inconsistent results under production conditions [52]. One promising method is the use of genetic resistance through resistant cucumber varieties, which can provide an economical solution alone or in combination with other control methods [51].

*Pythium Fruit Rot* is caused by various fungus-like organisms belonging to the genus *Pythium* [6]. Initial symptoms include water-soaked, brownish lesions that rapidly expand, becoming large, watery, soft and rotten [6]. This type of rot typically affects the parts of the fruit that are in direct contact with the soil [6,53]. Often, a brown to dark green blister can be seen on the cucumber fruit before it turns watery and begins to decompose and finally die [6,54]. To control *Pythium*, chemical fungicides, temperature and moisture control, compost use and biological control strategies are emphasized [55]. Chemical fungicides such as *Metalaxyl* and *Azoxystrobin* are common, but their effectiveness varies by crop and *Pythium* species [55]. Biological approaches, such as certain bacterial strains, show promise in suppressing *Pythium* colonization and promoting plant growth [55,56]. The use of microorganisms that suppress the main causes of *Pythium* has already been used successfully on pepper plants [56]. A combination of these methods could provide a more effective strategy for controlling *Pythium*-related diseases [55].

*Gummy Stem Blight*, a fungal infection, impacts cucumber plants by producing brown or grayish-brown lesions on the leaves that may appear gummy or sticky [6]. This disease can significantly affect the plant’s growth and yield by causing leaves to wilt and eventually drop off [6,57,58]. In severe cases, the fungus can cause the stem of the cucumber plant to rot and break down, leading to the complete death of the plant [6]. The treatment of *Gummy Stem Blight* in cucumbers can be achieved through the development and use of resistant cucumber varieties [59]. This reduces the use of fungicides, which also has a positive effect on environmental and food safety risks [59]. Another option is the use of plant growth-promoting fungi [60]. This variant not only effectively treats the disease and boosts plant growth but also represents an efficient and environmentally friendly strategy [60].

Cucumber plants can fall prey to *Downy Mildew*, a widespread fungal condition, particularly in wet or humid climates [6,61]. Affected cucumbers display yellow or light green spots or blotches on the top side of their leaves, accompanied by a downy growth on the leaf’s underside, which can appear white or grayish-purple [6,61]. The aﬄicted leaf areas might form lesions that expand and merge, causing the leaves to turn brown or die off [61]. Leaves may also curl or become misshapen, ultimately drying up and detaching from the plant [6]. The treatment of *Downy Mildew* involves the use of various fungicides, including Oxathiapiprolin, which is highly effective against the pathogen Pseudoperonospora Cubensis [62,63]. Fungicides are selected based on their ability to reduce symptoms and protect yields [63]. Resistance to some fungicides has been observed, emphasizing the need to alternate fungicides with different modes of action to slow the development of resistance [62,63].

### Deep learning in agriculture

Advancements in machine learning and computer vision have led to the development of new approaches for automating plant disease recognition through image processing techniques. Significant progress has been made in identifying and diagnosing leaf diseases in cucumbers and other plants using traditional machine learning methods such as support vector machine, AdaBoost, k-nearest neighbor and probabilistic neural networks (PNNs) [64–67]. Recently, deep learning-based algorithms have become dominant in image classification and recognition. They offer promising results and superior potential for feature extraction and recognition by processing input data through multiple non-linear functions, outperforming traditional shallow methods. Deep learning models, in particular, have demonstrated a significant amount of potential for the automatic identification and categorization of plant diseases and have shown remarkable superiority over traditional methods [30,68–70]. Numerous papers, previously introduced, use transfer learning for image recognition tasks [7,14,71].

Transfer learning is a well-established method in machine learning. Pre-trained models, developed using extensive datasets, are employed to train additional models that have limited training data available [72]. Traditionally, in transfer learning, the lower layers of a pre-trained network, such as VGG-19, are frozen to leverage the generalized features that have been trained on broad and diverse datasets like ImageNet [73]. Only the last layers, which are specifically added for the new task, undergo fine-tuning [74].

During the fine-tuning process, the previously frozen layers are gradually unfrozen. These layers are then trained at a reduced learning rate to allow fine adjustments to the features without disrupting the already learned and useful representations [75]. This method maximizes the utilization of the pre-trained network architecture and prevents excessive specialization on the training datasets, potentially enhancing the model’s generalization capabilities [73].

### Related work on cucumber disease detection

As a result, numerous studies have been dedicated to identifying cucumber diseases using machine learning techniques. Table 1 presents a summary of these studies and provides an overview of the related work.

Some studies focus on determining the severity of a disease on a cucumber leaf.

Lin et al. [31] utilized a semantic segmentation model based on a convolutional neural network (CNN), inspired by the U-Net architecture, to classify the cucumber leaf disease *Powdery Mildew* on cucumber leaves, using a dataset consisting of 50 images of affected cucumber leaves taken under laboratory conditions. Their model segments *Powdery Mildew* on cucumber leaf images at the pixel level, enabling precise quantification of the infected areas. Lin et al. achieved an accuracy of 96.08%.

Ozguren [32] aimed to contribute to the detection of *Mildew disease* and determination of the disease severity using cucumber leaf images. A dataset with a total of 175 images was used to classify the extent of the disease into five classes. The author utilizes image processing techniques to enhance feature extraction within the images. A Faster R-CNN model was used to achieve an accuracy of 94.84%.

Bansal et al. [30] investigated the cucumber leaf disease "cucumber leaf spots" by the severity categorized into 5 classes. The used dataset contains 50,000 cucumber leaf images. The deep learning model ResNeXt50 was applied to this dataset, resulting in an accuracy of 97.81%. ResNeXt50 is a deep CNN that builds upon ResNet but introduces grouped convolutions, improving model efficiency and accuracy.

Other studies focus on the recognition of different cucumber leaf diseases.

Zhang et al. [33] proposed an advanced CNN for the recognition of three different cucumber leaf diseases. The used dataset contained images of cucumber leaves taken in the field. They employed a two-stage segmentation method combining GrabCut and Support Vector Machine algorithms to extract disease spots from cucumber leaves. This approach utilized color, texture, and border features to accurately segment lesion areas from images captured in field conditions. Zhang et al. [33] achieved an accuracy of 96.11%.

Hussain et al. [28] used a fusion of two deep learning models, VGG and InceptionV3, to detect five different cucumber leaf diseases. With the self-collected dataset, an accuracy of 96.50% was achieved. The authors focused on feature extraction and automatic feature selection in their work. First, they extracted features like color, texture, and shape descriptors. Then they identified the most relevant features, which they subsequently used for classification.

Khan et al. [34] compared four deep learning models: VGG16, ResNet50, ResNet101 and DenseNet201. The used dataset aimed to distinguish six different cucumber leaf diseases. By using DenseNet201, an accuracy of 98.40% was achieved. The authors also used automatic feature selection with a technique called Entropy-ELM. That technique allows to retain the most informative features while eliminating redundant or irrelevant ones.

Ma et al. [7] compared traditional methods, random forest and support vector machine, with a deep CNN. With a large dataset of 14,208 images, four cucumber leaf diseases were detected. The deep CNN achieved an accuracy of 93.40%.

Omer et al. [35] built a CNN model from scratch to detect five cucumber leaf diseases with an accuracy of 98.19%. In their work, the self-designed CNN is compared with three other, pretrained models (AlexNet, InceptionV3, ResNet50), using a dataset consisting of 4,868 images. The proposed CNN model consists of five convolutional layers, partly followed by batch normalization and ReLU activation functions. Max-pooling and dropout layers are interspersed. The network concludes with two fully connected layers, culminating in a SoftMax output layer.

Nguyen & Nguyen [36] utilized a recurrent residual U-Net deep learning model for feature extraction in their approach. Then a support vector machine classifier is used with the extracted features for classification. For the recognition of four different diseases, the authors achieved an accuracy of 96.33%, using a dataset of 154 images.

Tani et al. [37] focused on the recognition of multiple infections on cucumber leaves. In their work, 25 classes with 11 different diseases are considered. 13 of the 25 classes show more than one infection of the cucumber leaves. With a CNN-based VGG-Net model, the authors achieved an accuracy of 95.50% for the entire dataset. The diseases of the cucumber leaves with multiple infections were detected with an accuracy of 85.90%.

Jeny et al. [38] focused exclusively on the recognition of diseases on the cucumbers and not on cucumber leaves. A random forest model was applied to a composite dataset from field images and open-source images. With an accuracy of 85.84%, five different cucumber diseases were classified.

Mia et al. [14] compared traditional machine learning methods with CNN-based transfer learning models for the recognition of diseases on cucumbers and cucumber leaves. The used dataset contains both photos from the field and photos under laboratory conditions. The six diseases were most accurately recognized with a MobileNetV2 with 93.23%. The most accurate traditional method was random forest with 89.93%.

Junior & Majiid [39] already addressed the same dataset for the detection of six different cucumber diseases, healthy cucumber leaves and healthy cucumbers. The models EfficientNetB0 and ResNet50 were used to achieve an accuracy of 94.20% using an averaging technique. However, the highest accuracy of 94.98% was reached using only EfficientNetB0.

Overall, the studies reviewed in this section highlight the effectiveness of ConvNets and other machine learning methods in detecting cucumber diseases. The related studies show very good results exceeding 90%. But Table 1 also shows, that studies that do not focus solely on the cucumber leaves but also on the cucumber itself produce poorer and inadequate results. Considering that, there’s a need for more comprehensive approaches that simultaneously address diseases affecting both cucumber leaves and the fruit itself, ensuring a holistic assessment of plant health.

Given the still promising results, we address this and develop an accurate model for the detection of cucumber diseases and cucumber leaf diseases exceeding the existing benchmark. As well, this study addresses several diseases within the same dataset, offering a more comprehensive diagnostic tool. This advancement not only allows for disease identification but also for assessing the overall plant health.

## Methodology

### Model architecture

The VGG19 model, originally introduced by Simonyan and Zisserman in 2014 [76], is a deep CNN comprising 19 layers. Trained on the ImageNet dataset, it excels in tasks such as image classification or object recognition [76]. For its input, the model necessitates an image size of 224x224 RGB pixels.

The model consists of five convolution blocks. These layers are responsible for extracting features from the input images. Equation 1 shows the mathematical equation of a 2-D convolution. During convolution, the kernel (w) is moved over the input image (x). A point (i,j) corresponds to a specific point in the output. The summation formula describes a loop over all positions (m,n) [77] .


$$\begin{array}{ccc} \left(y=x*w)[i,j]=\underset{m}{\sum }\underset{n}{\sum }x[i-m,j-n]w[m,n\right] & & \end{array}$$ (1)

Subsequently, to each convolutional block, a max-pooling layer is applied to downsample feature maps and reduce their spatial dimensions. The fully linked layers use these features to execute the final classification or recognition. Notably, the VGG19 model employs very small 3×3 convolutional filters and can contain up to 512 filters in each layer. The Rectified Linear Unit (ReLU) function is implemented to activate the hidden layers [76], facilitating the model in learning complex features from input images. Furthermore, the deep network architecture allows the model to capture hierarchical representations of the input images.

The selection of the VGG19 model for image recognition is justified by its architecture, which uses small convolution filters to deepen the network without greatly increasing parameters [76]. This design enhances its ability to differentiate features and improve generalization through implicit regularization. Demonstrating effectiveness in various large-scale image recognition studies [78,79], VGG19 also serves as a robust base for transfer learning, adapting well to new datasets by adjusting pretrained weights to meet specific application needs [80].

**Fig 2:**
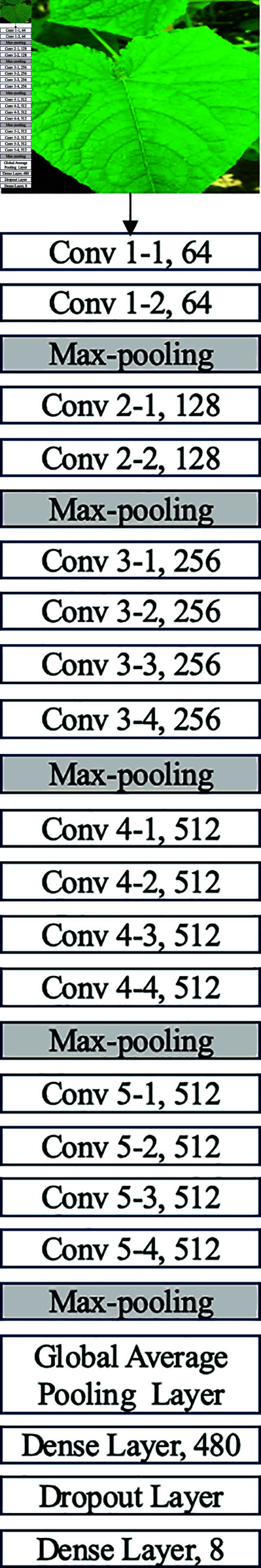
Our model architecture.

Our model modifies the VGG19 base up to the last max-pooling layer. We added a global average pooling layer to reduce overfitting and convert feature maps to a one-dimensional vector for processing by the dense layers [81]. The network then includes a dense layer with 480 neurons and ReLU activation, followed by a 20% dropout layer to further prevent overfitting. The final dense layer has eight neurons with softmax activation, configuring the model for specific recognition tasks. The architecture of the model is shown in Fig 2.

### Process of training

We trained the model with different hyperparameters which we identified by using the Hyperband tuner, a powerful and efficient hyperparameter optimization strategy provided by Keras. Hyperband offers several advantages, including its efficient resource allocation, which results in faster convergence to optimal configurations [82]. It dynamically allocates resources to more promising configurations through its unique bracketing and halving approach, leading to significant time savings and improved performance in machine learning model training [82]. During the process, we tuned the hyperparameters batch size, dropout rate, learning rate and the number of units for the first dense layer. The batch sizes of 16 and 32 were evaluated, the learning rates of 0.001, 0.0001 and 0.00001 were considered, the dropout rate ranged from 20% to 50% and the number of units for the dense layers varied between 192 and 512.

Batch size, the count of samples processed by the model in a single iteration, was set to 16. The number of epochs, meaning the amount of times the entire training data set was passed through the model during the training process, was 53 after a callback function was triggered. This callback was activated after 20 epochs without any improvement in the validation loss. The learning rate determines the size of the steps that a model takes during training to adjust its weights and was 0.00001. We employed the Adam optimizer [83] algorithm as a stochastic optimization method. It is used for first-order gradient-based optimization of stochastic objective functions, leveraging adaptive estimates of lower-order moments [83].

Our approach involved the utilization of a VGG19 model that had previously been pretrained on the ImageNet dataset [76]. We initiated the model customization by first removing its top layer. We subsequently froze the weights of the first eleven layers to ensure their preservation during training, leaving the last eight layers adjustable. Considering the depth of the VGG19 model, we wanted to retain the knowledge encoded in the earlier layers while allowing the later layers to be adapted to our dataset. This approach strikes a balance between leveraging the general feature extraction capabilities of VGG19 and tailoring the model’s learned representations to our specific eight-class cucumber disease recognition task.

To adapt the model for our specific task of cucumber disease recognition, we introduced new layers into the architecture. These additional layers encompassed a global average pooling layer introduced by Lin et al. [84], designed to reshape the extracted features, a dense layer comprising 480 units and employing the ReLU activation function to capture high-level features, a dropout layer randomly dropping 20% of the neurons that are deactivated during training and an output layer featuring eight units, each corresponding to one of the eight classes within our dataset. These newly introduced layers were seamlessly integrated into the existing model, effectively replacing the previously excluded top layer.

For the traditional transfer learning approach, the hyperparameter tuning was nearly equivalent. Batch sizes of 16 and 32 were tested, learning rates of 0.1, 0.01, 0.001, 0.0001 and 0.00001 were selectable, dropout rates ranged from 0.2 to 0.5 and the number of units for the dense layers spanned from 96 to 512. The final model was trained with a batch size of 16, a learning rate of 0.001, 288 units for the dense layer and a dropout rate of 30%. The model underwent 87 epochs of training, with fine-tuning beginning after 50 epochs. Additionally, the learning was successively reduced by a factor of 10 until it reached 1e-9 at the end. For both the initial training process and fine-tuning, the same callback function as in the novel approach was employed but only triggered during the fine-tuning process.

### Novel transfer learning approach

In comparison to ordinary fine-tuning approaches, our approach significantly modifies this traditional methodology by making selective layers, in our case the last eight, of the VGG-19 model adjustable from the beginning of the training process. This method enables direct and intensive use and adaptation of the deeper layers of the network for the specific task of disease detection in cucumbers [85]. Immediate adjustment of these layers allows the model to swiftly respond to the complex and specific features of the target images, which is particularly advantageous when dealing with limited datasets [85].

This strategy offers potential benefits in terms of adaptation speed and the model’s responsiveness to new data [86]. By making critical layers trainable from the beginning, the flexibility of the model to react to subtle differences in the training data and effectively utilize them is enhanced [86]. This approach could prove particularly advantageous in scenarios where specific features, not adequately represented in broad training datasets, are crucial. While this less conventional method may heighten the risk of overfitting, proper management and calibration of the model could ensure that the benefits of a quicker and more focused adaptation significantly outweigh the potential risks. This paper compares both the traditional fine-tuning approach and our novel transfer learning method. We analyze the performance, adaptability and efficiency of each approach in the context of disease detection in cucumbers.

### Evaluation method

During the model training the performance metrics loss and accuracy were calculated using the validation dataset.

After the model training we evaluated it by measuring the performance indicators (2)-(7). The equations for accuracy, recall, precision and F1 score are derived from Sokolova and Lapalme [87]. Balanced accuracy as a metric is based on Brodersen et al. [88]. This measure is especially important when dealing with imbalanced datasets where it provides a more realistic evaluation by considering both sensitivity and specificity [88]. It’s essential for ensuring that the performance improvements are not skewed by any class imbalance within the dataset.

In a multi-class scenario involving eight categories, True Positives (TP) refer to correctly identified instances belonging to a specific class, while True Negatives (TN) represent accurately recognized instances not belonging to that class [87]. False Positives (FP) occur when instances are incorrectly labeled as belonging to a certain class and False Negatives (FN) arise when instances are incorrectly labeled as not belonging to that class [87]. These metrics are crucial for assessing and calculating the performance of classification models consisting of the accuracy of the test dataset, precision, recall and F1 score [87].

The test accuracy provides an overall view of model correctness, while the precision focuses on the accuracy of positive predictions, recall assesses the model’s ability to find all positive instances and the F1 score offers a balanced metric that considers both precision and recall. We also present a confusion matrix to further evaluate every class within the dataset.


$$\begin{array}{ccc} Accuracy=\frac{\text{TP + TN}}{\text{TP + TN + FP + FN}} & & \end{array}$$ (2)

$$\begin{array}{ccc} Precision=\frac{\text{TP}}{\text{TP + FP}} & & \end{array}$$ (3)

$$\begin{array}{ccc} Recall=\frac{\text{TP}}{\text{TP + FN}} & & \end{array}$$ (4)

$$\begin{array}{ccc} Specificity=\frac{\text{TN}}{\text{TN + FP}} & & \end{array}$$ (5)

$$\begin{array}{ccc} F1Score=\frac{2\;\times \;\text{Recall}\;\times \;\text{Precision}}{\text{Recall}+\text{Precision}} & & \end{array}$$ (6)

$$\begin{array}{ccc} BalancedAccuracy=\frac{\text{Recall}+\text{Specificity}}{\text{2}} & & \end{array}$$ (7)

### Optical model evaluation

To enhance the explainability of our model after prediction, we looked at the "output" images of our model. Therefore we utilized Local Interpretable Model-agnostic Explanations (LIME), which is a method that generates local, easy-to-understand explanations for individual predictions of models such as deep neural networks.

LIME explains predictions from complex “black-box” models by approximating them locally with simpler, interpretable models. To explain a single instance, LIME creates new, slightly perturbed samples around that instance and obtains predictions from the black-box model for each perturbed sample. Next, it trains a simple surrogate model—often a sparse linear classifier or small decision tree—using these perturbed samples and their corresponding predictions, while weighting points closer to the original instance more heavily. Because this surrogate model is easy to interpret, its coefficients or rules reveal which features most strongly influence the prediction in the instance’s local neighborhood. By focusing on neighborhoods rather than the entire data space, LIME delivers accurate local explanations without requiring knowledge of the black-box model’s internal architecture [89]. The output of LIME is therefore a heat map that shows the areas of the input image on which the applied model makes its decisions.

**Fig 3:**
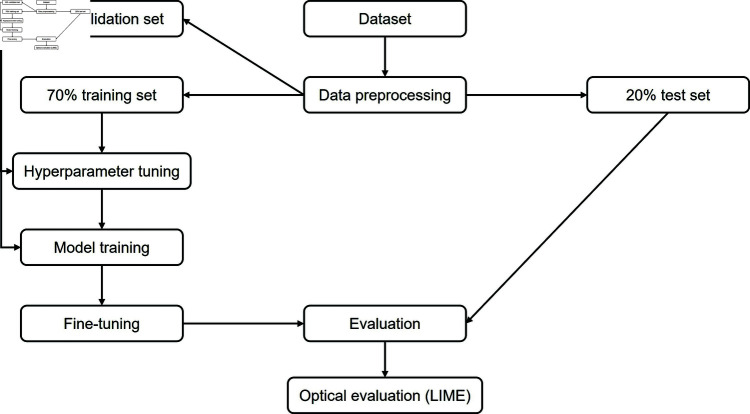
Methodical procedure.

Fig 3 shows the complete methodical procedure that we applied in this work.

### Data preprocessing

During data preparation 1,280 images were resized to 224 x 224 pixels, labeled according to their class name and scaled to a range of [0, 1] for normalization using the rescale parameter with a value of 1/255. To assess the model’s performance, a hold-out cross-validation methodology was employed. We randomly divided the dataset into training (70%), test (20%) and validation (10%) subsets to mitigate the risk of overfitting to specific image sequences. We utilized the training subset to train the model, while performance metrics were computed using the validation subset following the training process. Importantly, the testing subset remained unseen by the model during the training phase and was solely employed to evaluate performance metrics after model training.

Next, we applied different data expansion techniques for the training set using various augmentation layers from Keras. Data augmentation is a pivotal technique employed to enhance the diversity of the training dataset and improve the generalization capability of deep learning models. In the context of this study, a comprehensive set of random transformations and operations was applied. This process introduces variations in the images and reduces the risk of overfitting.

**Fig 4:**
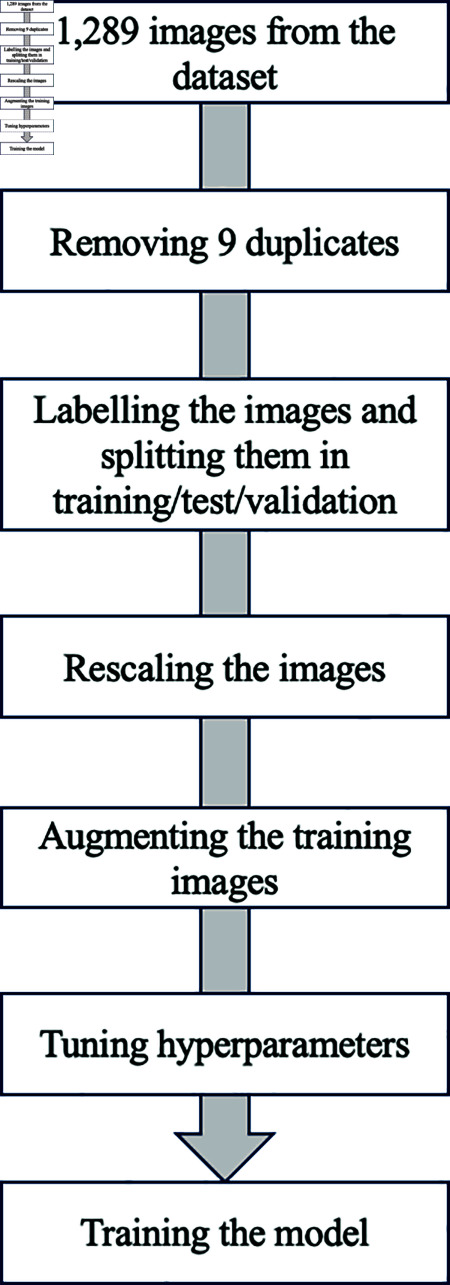
Data preprocessing.

The operations encompassed random horizontal and vertical flips and rotations of 30% in each direction. Zooms ranged between 75 to 125% from the original image size. The width was up to 20% in expanding as well as 20% in narrowing. In addition, the brightness and contrast of the images were up to 25%. Changes in height and translations varied between -15 to 15%. Collectively, these augmentations served to introduce variations and diversity into the training data, thus mitigating overfitting risks and enhancing model robustness. The complete data preprocessing steps are illustrated in Fig 4.

### Dataset

**Fig 5:**
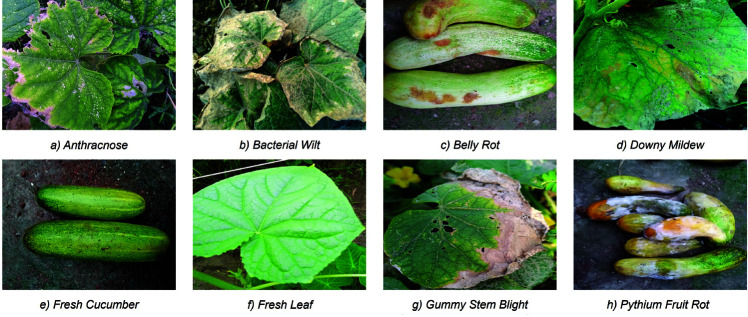
Exemplary images from the classes of the dataset.

The dataset we used includes 1,289 RGB images and is provided by Sultana et al. [6]. Fig 5 shows the eight types of cucumber classes, namely *Anthracnose, Bacterial Wilt, Belly Rot, Downy Mildew, Pythium Fruit Rot, Gummy Stem Blight, Fresh Leaf* and *Fresh Cucumber*. Each class consists of 160 images and is organized in a separate folder. The folder with images of *Pythium Fruit Rot* contains 169 images, including nine duplicates that we removed during the data preprocessing. The dataset is freely available on the Mendeley website.

The data was collected from a field in Basundia in Bangladesh under natural weather with inconsistent lighting conditions [6]. The images were captured in the size of 512 × 512 pixels using a Nikon D5300 digital camera with the cooperation of an expert from an agricultural institution [6].

## Results

In the evaluation of unseen test data, the model demonstrated an accuracy rate of 95.31% while the traditional approach reached an accuracy of 87.89%. Prior efforts by Junior and Manjid [39] concerning this dataset achieved an accuracy of 94.20% with a model leveraging the combination of ResNet50 and EfficientNetB0 and 94.98% by using a technique solely based on EfficientNetB0.

**Fig 6:**
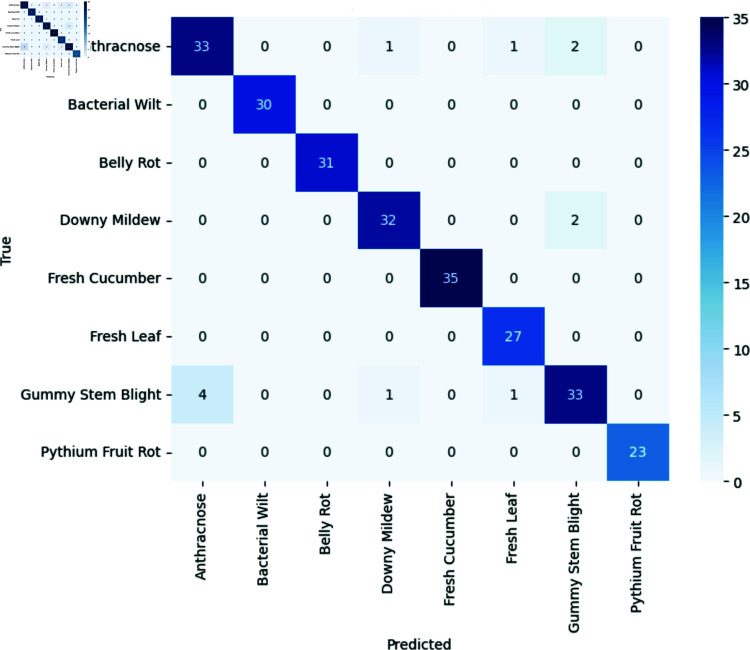
Confusion matrix of the novel approach.

**Fig 7:**
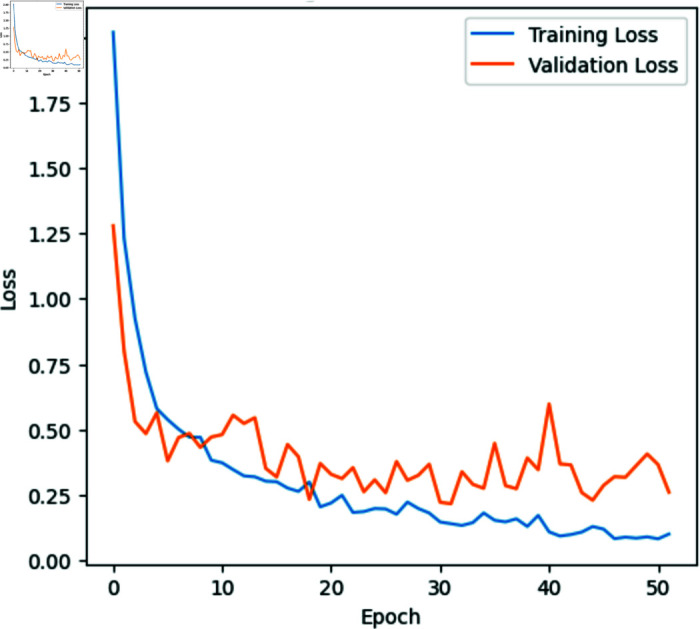
Training and validation loss of the novel approach during the training period.

**Fig 8:**
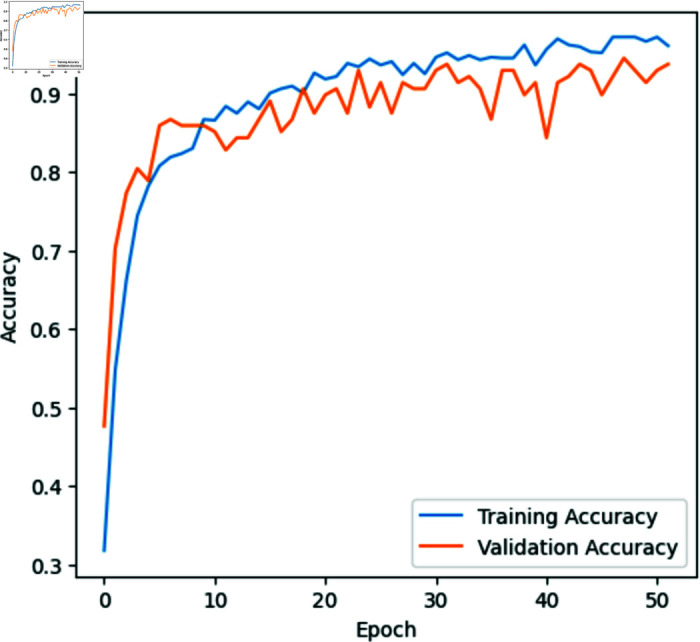
Training and validation accuracy of the novel approach during the training period.

With a recall of 95.99%, our model reliably identified 95.99% of the dataset’s classes. Moreover, the model exhibited a precision of 95.70%, signifying its ability to correctly classify images with a high degree of accuracy, at a rate of 95.70%. The calculated F1 score, standing at 95.85%, serves as an indicator of the model’s balanced performance in terms of precision and sensitivity. Notably, our model achieved a balanced accuracy of 97.66%, surpassing the traditional approach reaching 93.87%. The performance for each class on unseen data is illustrated in the confusion matrix in Fig 6 and Table 2. The model’s accuracy and loss during the training period are represented in Figs 7 and 8.

**Table 2:** Performance metrics for each class: novel approach / traditional approach.

Class	Images	Precision, %	Recall, %	Specificity, %	F1 score, %	Balanced accuracy, %
Anthracnose	37	89.19 / 66.67	89.19 / 81.25	98.16 / 94.20	89.19 / 73,24	93.68 / 83.98
Bacterial Wilt	30	100 / 93.55	100 / 96.67	100 / 99.12	100 / 95.08	100 / 97.89
Belly Rot	31	100 / 100	100 / 96.77	100 / 100	100/ 98.36	100 / 98.39
Downy Mildew	34	94.12 / 87.88	94.12 / 85.29	99.10 / 98.20	94.12 / 86.57	96.61 / 91.75
Fresh Cucumber	35	100 / 97.22	100 / 100	100 / 99.55	100 / 98.59	100 / 99.77
Fresh Leaf	27	93.10 / 67.50	100 / 100	99.13 / 94.47	96.55 / 80.60	99.56 / 97.23
Gummy Stem Blight	39	89.19 / 81.25	84.62 / 66.67	98.16 / 97.24	86.91 / 73.24	91.39 / 81.95
Pythium Fruit Rot	23	100 / 100	100 / 100	100 / 100	100 / 100	100 / 100
Overall	256	95.70 / 86.76	95.99 / 90.83	99.32 / 97.85	95.85 / 88.21	97.66 / 93.87

## Discussion

Fig 6 as well as the test accuracy of 95.31% and balanced accuracy of 97.66% indicate the performance of the model. It accurately predicts whether a cucumber or its leaves are healthy or diseased with *Anthracnose, Bacterial Wilt, Belly Rot, Downy Mildew, Pythium Fruit Rot* or *Gummy Stem Blight*.

The observed outcomes can largely be attributed to the inherent complexity of the VGG19 model. As a deep CNN architecture, VGG19 possesses a profound depth and intricate layering, enabling it to effectively learn intricate features from input images. This complexity empowers the model to discern subtle variations crucial for accurate disease recognition, as it captures intricate patterns and nuances within the data. Additionally, the hierarchical structure of VGG19 facilitates the progressive extraction of features at different levels of abstraction, ranging from simple edges and textures to more complex shapes and structures. Consequently, the robust performance demonstrated by the VGG19 model underscores the critical importance of leveraging sophisticated deep learning architectures in addressing the challenges inherent in plant disease detection and classification within scientific research contexts.

The progression of training and validation metrics, as depicted in Figs 7 and 8, reveals significant insights into the model’s learning behavior and the effectiveness of the early layer unfreezing strategy in comparison to traditional training approaches.

The rapid improvements in the initial iterations can be attributed to the model quickly learning the high-level features necessary for disease identification in cucumbers, which are crucial for initial gains in accuracy and loss reduction. This initial surge is typically seen in models where higher layers have been focused during fine-tuning, allowing the model to efficiently adapt to the specifics of the task.

The subsequent quick stabilization of the model after approximately 20 to 30 epochs suggests that the major, distinguishable features of the dataset have been effectively captured. The early unfreezing of several last layers during hyperparameter tuning likely contributes to this early plateau, as the foundational visual patterns do not undergo further modifications, thus maintaining stability in feature extraction across the training process. This stabilization is a desired outcome, indicating that the model is not overfitting excessively to the noise within the training data, which can be a common pitfall in deep learning models without such layer constraints.

In contrast, the traditional approach where layer unfreezing is implemented later in the training phase leads to different dynamics. It’s showing signs of overfitting as early as epoch 10. This is typically evidenced by a stagnation or decline in validation accuracy, even as training accuracy continues to rise. This divergence between training and validation performance suggests that the model is becoming too specifically tuned to the training data, losing its ability to generalize to new, unseen data. The minimal improvements in validation accuracy post-epoch 10 in such traditional setups may indicate that while the model continues to optimize based on the training set, it fails to provide additional value on validation or real-world datasets.

Table 2 and Fig 6 illustrate the individual performance of each class within the test dataset. Our model demonstrated perfect recall (100%) in identifying fresh cucumbers and leaves, although misclassifying two diseased cucumber leaf images as *Fresh Leaf*. Moreover, it exhibited high accuracy in predicting the diseases *Bacterial Wilt, Belly Rot* and *Pythium Fruit Rot* with F1 scores of 100%, while *Downy Mildew* reached a F1 score of 94.12%. However, the model encountered challenges in correctly classifying Gummy Stem Blight, misidentifying four images as *Anthracnose* and two images as either *Bacterial Wilt* or *Fresh Leaf*. Conversely, images depicting leaves aﬄicted with *Anthracnose* and *Downy Mildew* were both twice erroneously labeled as Gummy Stem Blight. Although, Fig 5 shows that there are some similarities between the three classes *Anthracnose*, *Bacterial Wilt* and *Downy Mildew* several factors may contribute to these discrepancies.

First, *Gummy Stem Blight* may possess visual characteristics or patterns resembling those of other classes in the dataset, leading to classification confusion. Additionally, noise or variability in the images, coupled with the complexity of the classification task, could contribute to misclassifications. Furthermore, the uneven distribution of classes in the test dataset, resulting from the random assignment of images, may potentially impact the model’s ability to accurately differentiate between them. Conversely, *Anthracnose* may exhibit more distinctive or easily recognizable features, facilitating correct classification and reducing the likelihood of misidentification as *Gummy Stem Blight*.

**Fig 9:**
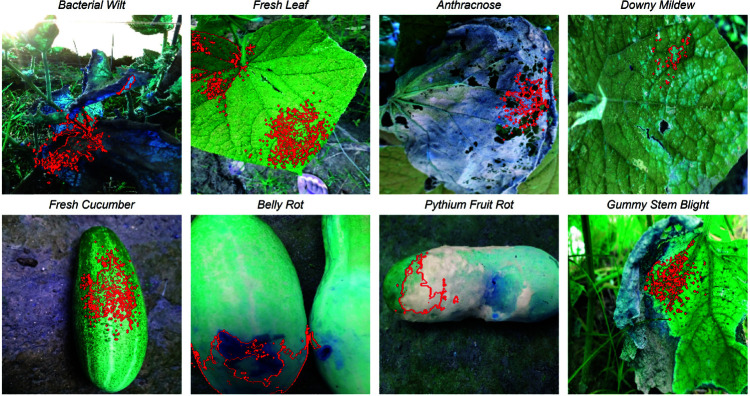
Exemplary LIME results.

In Fig 9 the results of the LIME application are shown. The output images of the algorithm show, for example, a reasoned decision for the respective prediction for each class. The examples show that in nearly each of these cases, our model focuses on the areas of the image that are actually relevant for recognizing the corresponding class, which suggests that the model has actually learned the relevant features from the relatively small dataset.

Also there is a possible explanation why the model could rarely recognize *Gummy Stem Blight* for *Anthracnose* or *Downy Mildew*. It can be seen that the model in the example does not concentrate on the affected parts of the leaf but on a healthy part, which could also fit the other two classes.

Table 3 sets the work we propose in relation to the most relevant existing research works. We selected those of the existing works that achieved the three best classification results. On the other hand, we have also chosen a work that examines both cucumbers themselves and their leaves.

**Table 3:** Comparison of Most Relevant Existing Work and this Study.

Reference	Year	Research Subject	Accuracy
Bansal et al. [30]	2023	Detection of severity level of cucumber leaf spot disease	97.81%
Khan et al. [34]	2021	Cucumber leaf disease recognition	98.40%
Omer et al. [35]	2022	Diagnosis of cucumber leaf diseases	98.19%
Junior & Majiid [39]	2023	Cucumber disease and cucumber leaf disease detection	94.98%
This work [39]	2023	Cucumber disease and cucumber leaf disease detection	97.66%

What can be seen directly in the table is that the works that only deal with the classification of cucumber leaves achieve better results than those works that also look at the cucumber itself. This could be because the leaves are similar and the cucumber represents a completely new object for the classifiers.

Nevertheless, it can be seen that our work can achieve competitive classification accuracies compared to these works and outperforms the best work dealing with leaves as well as the cucumber itself by almost 3%, setting a new benchmark for this specific problem.

Overall our model demonstrates very high accuracy with images captured under natural weather conditions and inconsistent light conditions. However, to enhance its real-world applicability, it needs to improve resilience against a broader range of environmental conditions such as extreme weather, varying growth stages and diverse plant species. In order to increase the model’s reliability, it is essential to consider additional validation and expert review for the cases where the model confuses *Anthracnose* and *Gummy Stem Blight* to minimize potential misdiagnoses. In addition, we propose the following Enhancements to enable applicability in agriculture:

The model should be integrated into existing agricultural management systems, enabling scalability from small farms to large-scale operations. This includes the potential for IoT-based applications that can respond dynamically to the diagnoses provided by our model.

To increase usability and acceptance, it is crucial to develop a user-friendly interface for farmers and offer comprehensive training programs. These programs will educate end-users on how to effectively utilize the system, ensuring that they can fully leverage its capabilities for better crop management.

By enabling early disease detection, the model can significantly reduce crop damage and decrease the reliance on pesticides. Highlighting these economic benefits will help demonstrating the cost-effectiveness of adopting this technology, which could lead to broader acceptance and implementation.

Strategies to promote technology adoption among farmers should be implemented. This could include demonstration projects, success stories and partnerships with agricultural advisors to showcase the benefits and practicality of the technology in real-world settings.

This would allow for early disease detection and timely intervention, significantly reducing crop damage and facilitating effective plant protection measures, thereby improving agriculture efficiency and sustainability.

## Conclusion

Automated, image-based detection of crop diseases is vital for managing crop health efficiently. Many existing classification systems struggle to handle the challenges posed by the irregular, complex and diverse characteristics of diseased cucumbers and cucumber leaves. As a result, there is a clear need for improved systems that can effectively recognize cucumber diseases automatically.

This paper showed a successful application of our new approach using a VGG19-based deep learning model to recognize healthy and diseased cucumbers. The results show that VGG19 is an effective CNN for this task, especially by proceeding after our approach. By freezing the last eight layers during fine-tuning, the VGG19 model therefore sets a benchmark in the detection of fresh cucumbers and the following cucumber (leaf) diseases including *Anthracnose, Bacterial Wilt, Belly Rot, Downy Mildew, Gummy Stem Blight* and *Pythium Fruit Rot*. The accuracy of our model (95.31%) in comparison to an ordinary approach (87.89%) suggests, that it has the potential to be a valuable tool for the agricultural industry, enabling more targeted use of pesticides or alternative techniques, helping to reduce the cost for the farmers and environmental harm by treating cucumber diseases.

### Limitations

While our proposed approach demonstrates promising results, it is important to acknowledge its limitations. Although we achieved high internal validity by employing a hold-out split, external validation of the model has not yet been conducted. To address this gap, additional testing of the algorithm on datasets comprising multi-class plant disease data is required. The generalizability of the model may also be constrained by the specific characteristics of the dataset used for training and evaluation. Variations in image quality, lighting conditions and disease severity across different datasets may affect the model’s performance. By visually evaluating the classification results, we have come to the conclusion that *Gummy Stem Blight* in particular is often classified incorrectly. The data set will be expanded to perhaps more comprehensively map this class in future research.

### Future work

To address the limitations identified in this study, several approaches for future research can be explored. Firstly, investigating the transferability of the trained model to diverse datasets or real-world scenarios is essential to enhance its generalizability. Due to the lack of existing datasets including cucumbers and cucumber leaves, more data needs to be gathered [6]. This analysis would provide valuable insights into the model’s robustness and adaptability across different environments and conditions. In this context, ensemble learning could be used to combine the findings of several models into a common model. In future research, we will train several models individually with the few existing datasets and then merge them using ensemble learning to achieve a unified robust model [90].

Furthermore, the exploration of ensemble learning techniques presents an exciting opportunity to boost the model’s performance. By combining predictions from multiple models, ensemble methods leverage diverse representations of the data, potentially leading to improved classification accuracy and robustness. Another related approach could be to directly analyze the video streams from, for example, the drones using combined architectures from Recurrent Neural Networks and CNNs, which has already yielded promising results in other domains [91].
